# Culture, ethics and clinical practice for intensivists managing end of life care: an Australian perspective

**DOI:** 10.1186/s40560-026-00860-z

**Published:** 2026-02-19

**Authors:** Ryo Ueno, Lucy Modra, Stephen Warrillow

**Affiliations:** 1 Department of Intensive Care, Austin Health, Melbourne, Australia; 2 Monash University, Melbourne, Australia; 3 University of Melbourne, Melbourne, Australia

## Abstract

Death and dying are significant and impactful, for individuals, families and broader society. For clinical teams working in the intensive care unit (ICU), caring for a dying patient and supporting their family are an important part of their professional role. Australian ICU practice has evolved over several decades to optimise end of life care, so that it is patient centred and adheres to accepted ethical standards as well the established legal framework. In addition to acquiring necessary technical skills, intensivists working in Australia must complete training in advanced communication as well as clinical ethics and are required to maintain competence in these domains for the duration of their professional lives. Important considerations for Australian intensivists managing end of life care include cultural humility, avoidance of assumptions, respectful curiosity, prioritising individual patient values and preferences, and the avoidance of non-beneficial treatments that may simply prolong dying or contribute to suffering. As well as having a legislated legal framework, Australia has endorsed national guidelines developed by relevant the specialist training colleges and intensive care professional societies.

## Introduction

This article provides an overview of end-of-life care for critically ill patients admitted to intensive care units in Australia. Medicine, death and dying involve the intersection of legal, cultural, and ethical considerations, as well as practical matters relating to clinical management. These elements and their interactions are considered from the Australian perspective, followed by an outline of how end of life care is generally approached in Australian ICUs.

Australia has a unique combination of factors relevant to intensive care practice which are summarised in Table 1 [1]. Legislation relating to medical practice and death in Australia was developed over 40 years ago and is relatively consistent across Australian jurisdictions. Public healthcare is funded by taxpayers and delivers high-quality universal healthcare to all citizens and permanent residents.

**Table 1:** Important attributes of the Australian context [1]

Domain	Key points
Legal	• Brain death and circulatory death are both legally defined • Clinicians are not obligated to provide non-beneficial treatment • Withholding and withdrawing treatment are considered legally equivalent • Legally binding Advanced Care Directives cannot be over-ridden by family
Ethical	• The Australian and New Zealand Intensive Care Society supports a distributive-justice approach in resource-intensive decisions [38].
Societal	• Strong emphasis on individual autonomy rather than family-centered decision-making • Many patients prefer to avoid burdensome treatments associated with high dependency (e.g., nursing home) • Death and dying are less openly discussed • Generally high trust in clinicians • Tax-payer funded universal health care provided to all Australian citizens and permanent residents
Training and systems	• Single national ICU training pathway with consistent end-of-life standards, supported by examinations and mandatory courses • Ventilation, vasopressors, and dialysis are not commonly managed in long-term facilities • All ICUs operate under a ‘closed’ model with strong emphasis on a multidisciplinary team-based approach to patient care • Most ICUs combine a broad case mix of medical and surgical patients, operating as adult or paediatric units • Most patients who die in ICU have treatment limitations or treatment withdrawn and receive palliative care provided by the ICU clinical team

Australian society is remarkably multicultural, with considerable diversity in language, religion, and ancestral background—nearly a third of Australians were born overseas [2]. Respect for medical professionals and trust in healthcare is generally high [3].

Professional practice and training in critical care are supported by two professional organisations. The Australian and New Zealand Intensive Care Society (ANZICS) was established in 1974 and has provided consensus led expert guidelines to support clinical practice at a national level for the last 50 years. The College of Intensive Care Medicine of Australia and New Zealand (CICM) became responsible for all specialist training of intensivists in 2010, replacing separate well-established formal training programs managed by the Royal Australian College of Physicians and the Australian and New Zealand College of Anaesthetists.

While our aim is not to propose practice recommendations per se, the description of Australian practice and the outline of how intensivists approach communication at the end of life may be of interest to those seeking to develop and implement processes suited to their own legal, cultural and social context.

### Clinical vignette

#### A hypothetical (but typical) case

*Mr A, a 70-year-old man with frailty and advanced chronic obstructive pulmonary disease, was admitted to the ICU with septic shock due to pneumonia. By day 10, his condition continued to deteriorate despite antibiotics, vasopressors, and mechanical ventilation, with a high risk of mortality and, at best, a prolonged recovery requiring transfer to a nursing home. His family recalled that he had repeatedly expressed a wish not to go to a nursing home, let alone live dependent on machines.*

*Discussions about goals of care were complex. Mr A’s daughter, joining remotely from overseas, struggled with the idea of “giving up” and requested ongoing treatment, including tracheostomy, while several staff expressed discomfort about continuing invasive support that seemed to prolong suffering. Following a multidisciplinary meeting with ICU and Respiratory consultants, a medical consensus was reached that ongoing ventilation was no longer consistent with Mr A’s values and was, therefore, non-beneficial.*

*The team was about to meet the family again.*

This vignette illustrates the process and challenges intensivists commonly face when managing end-of-life care—balancing legal obligations, ethical principles, and societal expectations. It will be revisited to illustrate how Australian intensivists integrate cultural, legal, and ethical considerations into practice.

### The context—cultural and societal context of dying in an Australian ICU

Serious illness, dying, and death are universally impactful across all cultures and societies [4]. Ways in which these phenomena are dealt with are influenced by complex interactions between cultural attitudes, religious beliefs, legal considerations, resource availability and societal expectations. While some attitudes to death and dying may be attributed to specific religions, language groups or ethnicities, it is important to note that these affiliations may not always accord with the preferences of an individual who may otherwise identify with such a group, and within such groups there are likely to be a range of beliefs and practices [5]. Australian society is multicultural—especially in urban centres—with many racial, ethnic, religious and language groups represented [2]. For Australian intensivists, interacting with patients and families who come from a very different background or belief system to their own occurs on a daily basis [6]. It has become well-recognised that cultural humility, respectful curiosity, avoidance of assumptions based on stereotypes and effective communication are essential to practising patient-centred care [7].

It is common for Australians to hold and express somewhat contradictory attitudes to death and dying. Death itself is seldom discussed, and contemporary Australian culture may sometimes feel “death denying” [8]. Most Australians are prepared to accept burdensome therapies to prolong life even when the chances of success are uncertain. It is also true, however, that many people hold strong preferences to avoid being kept alive in circumstances where they are highly dependent on care for activities of daily living and are unable to interact with family members or exercise autonomy over decisions affecting themselves. While certainly not universal, it is common for individuals to express serious reservations about living in a nursing home facility [9], although such arrangements may be viewed as acceptable when the need becomes more proximate [10]. All Australian jurisdictions provide a legal framework for documented advanced care directives that are often based on either goals and values, specific instructions or a combination of these approaches. Despite being available for many years and supported by government funded public education campaigns for over 20 years, [11, 12] overall engagement remains fairly low and the majority of critically ill patients in Australian hospitals do not have a documented advanced care directive [13, 14]. It is increasingly recognised by clinicians and Australian government agencies that harmonising laws across jurisdictions, educating healthcare workers, and improving public awareness of advanced care planning is necessary to further improve engagement with these processes [11, 15–18].

### The framework—legal framework for end-of-life care in Australia

#### Legal definition of death

All Australian jurisdictions have a legal framework that provides a definition of death, as well as determinations relating to issues that such as consent, refusal of treatment, the withdrawal or non-commencement of non-beneficial treatments and palliative care. While relevant law varies slightly in some minor respects between states, there are important elements that are rather consistent. In Australian law, death is generally defined as either irreversible cessation of circulation of blood in the body of the person or irreversible cessation of all function of the brain of the person [19]. No jurisdiction attempts to codify precisely how such determinations are made, with the specific processes and requirements necessary to diagnose death instead being outlined in authoritative guidelines developed by specialist professional medical organisations. Consequently, while the legal definition of death may not substantially change over time, the accepted methods of making this important diagnosis may evolve in accordance with professional recommendations and technological advances. While some of the key principles, processes and the associated legal framework were developed and codified to support organ donation for transplantation, such considerations apply equally to circumstances where organ and tissue donation is not being considered or will not occur.

#### Patient autonomy and refusal of treatment

Individual patient autonomy is afforded considerable protection under Australian law; an informed competent adult, or their recognised surrogate decision maker, can refuse almost any treatment, even if this act will result in serious harm or death [20]. Support for individual autonomy even extends to voluntary assisted dying (VAD, sometimes referred to as medically assisted dying or euthanasia), which has been legalised in most Australian jurisdictions in response to widespread (but not complete) community support. While specific details vary somewhat, all states where the process exists only allow access to VAD under prescribed circumstances and require many mandated steps that must be strictly adhered to. These legal requirements mean that VAD almost never impacts intensive care clinical teams, and the relevant laws have little if any direct relevance to critical care practice. The presence of VAD as a legally supported process reflects values and attitudes across a broad cross section of Australian society [21].

#### Limits of autonomy: non-beneficial treatment

There are very few instances in which clinicians can insist on treatment against the expressed wishes of a competent patient; family members can also refuse an intervention if they believe it would not be in accordance with the person’s known wishes. Notably, this high level of autonomy does not confer the ability of a patient or recognised decision maker to insist on treatment that is, in the consensus opinion of the treating medical specialists, non-beneficial, harmful, or unlawful. By way of example, a family could refuse potentially lifesaving surgery for a serious underlying emergency condition in frail elderly relative if they believe that it would not be consistent with that person’s goals and values. However, they cannot compel the provision of non-beneficial surgery if, in the consensus opinion of appropriately qualified senior doctors, the surgery will not provide meaningful benefit or will cause harm. Importantly, families cannot generally refuse the provision of analgesia or similar measures if clinicians hold a firm professional belief that the patient is suffering and does not have capacity to decline such measures [22]. While such instances are extremely rare in clinical practice, healthcare professionals can legally provide necessary measures to alleviate genuine suffering in patients without capacity to decide for themselves, even if surrogate decision makers are not in agreement.

#### Withholding versus withdrawing treatment

In most Australian jurisdictions the law supports the widely accepted ethical principle that withdrawing a non-beneficial therapy is equivalent to not commencing the same treatment [23]. It is worth noting that while this equivalence is well-established in Australian practice that it may feel psychologically challenging for some clinicians and families to cease a therapy once it has commenced. This is especially true for treatments, such as mechanical ventilation that may successfully “defer death” for a short period of time, but do not in themselves offer a definitive path to meaningful recovery. Nonetheless, this legally supported principle provides patients, families and clinicians with the opportunity to initiate complex interventions in situations of uncertainty and observe the response, with the option of ceasing the therapy if the desired improvement fails to eventuate. Clinicians can generally trial a treatment for a period without being fearful that they “cannot stop what has been started” later [24].

#### Disability, discrimination, and ethical boundaries

Australian law places high value on the right for individuals to make decisions and choices regarding their healthcare. Discrimination based on disability or other protected attributes such as race, sex, age, gender identity, and religion is unlawful in all Australian jurisdictions [25]. Accordingly, doctors may not base decisions to limit treatment solely on the presence of disability. It is important therefor to avoid conflating disability with the presence of a progressive life limiting medical condition. This can sometimes cause confusion and uncertainty for patients, families and clinicians in circumstances where a patient has a serious underlying condition that results in both disability and progressive life-limiting medical problems. Under such circumstances, it may be ethically appropriate and legally permissible to limit treatment to avoid non beneficial care when the medical condition is not likely to meaningfully improve in response to a proposed intervention.

#### Surrogate decision-making

In situations where a person cannot make decisions for themselves due to illness, Australian law outlines a process, whereby an appropriate and available surrogate decision maker can act on that person’s behalf. While such legal structures vary somewhat between jurisdictions, all generally outline a hierarchy of available family members and significant others to assume this role in the absence of an existing documented surrogate decision maker. Previously, laws generally required such a person to make decisions that were based on a combination of medical recommendations and the “best interest” of the patient. Contemporary versions of these laws now mostly require that the surrogate decision maker must act in accordance with what they genuinely believe would be the decision that the patient would make for themselves if they were in a position to do so, even if that decision might somehow not otherwise be considered to meet the “best interest” test [22]. This is a subtle yet important difference, as it requires family members to make the decision that they honestly believe the patient would prefer rather than the decision that they themselves might want for their loved one. While these two perspectives will usually be well-aligned, there can be instances, where a family might wish to pursue aggressive treatment but decide not to do so based on knowing that their relative had previously expressed preferences to avoid such interventions.

### The practice—what happens in Australian ICUs?

#### Communication skills training for intensivists

Intensivists are required to communicate skilfully, often in high pressure and emotionally charged situations. Everyday examples include breaking bad news to a patient’s family, leading resuscitation teams, or negotiating treatment plans with other medical specialists. Excellent communication skills are considered a core attribute of an Australian intensive care specialist [26–28].

Intensive care specialty training through the College of Intensive Care Medicine of Australia and New Zealand (CICM) program incorporates mandatory training in communication skills, fundamentals of medical ethics, cultural humility, organ donation and high-quality end of life care. Once qualified, intensive care specialists are required to participate in a continuing professional development program that incorporates culturally safe practice, approaches to addressing health inequities, professionalism and ethical practice [29].

The College of Intensive Care Medicine, the Australia and New Zealand Intensive Care Society (ANZICS), and the Australian College of Critical Care Nurses (ACCCN) publish statements to guide best-practice communication and end-of-life care in the setting of critical illness, further underscoring the high value placed on end-of-life care in Australian intensive care practice. These publications include the ANZICS Statement on Death and Organ Donation, the ANZICS Statement on Care and Decision Making at the End of Life for the Critically Ill, and the joint CICM/ANZICS statement on withholding or withdrawing treatment in the setting of critical illness [23, 24, 30, 31].

### Approach to communication with ICU patients and their families

All ICUs in Australia operate under a ‘closed’ model that is led by intensivists who work closely with senior specialists from other treating unit to manage patients admitted to the ICU [32]. Patient care is delivered by a multi-disciplinary team including doctors and nurses who have completed formal post graduate qualifications in critical care. Allied health specialists with expertise in critical illness are integral to Australian ICU practice, with physiotherapists, dietitians, pharmacists, speech therapists, social workers, and chaplains all contributing to patient care [33]; however, psychologists are not directly incorporated into most units [34]. Intensivists aim to communicate regularly with the families of critically ill patients to build trust and rapport, improve the family’s understanding of the critical illness, and to establish their own understanding of the patient’s psychosocial context, goals and values.

When a patient requires an unplanned or emergency admission to the intensive care unit, Australian intensivists routinely contact their family or ‘next of kin’ to update them about their relative’s illness and planned treatment. This initial conversation with the patient’s family establishes trust and rapport with family members who may be surprised and distressed by their relative’s illness. Intensivists aim to speak plainly about the severity of the situation, conveying that illnesses requiring unplanned admission to the ICU are serious and can even be life-threatening. Discussion includes conveying what may be considerable prognostic uncertainty in the early phase of critical illness, acknowledging that this can be extremely stressful for family members.

These early discussions are helpful regardless of the patient’s outcome. For families of patients who recover from their illness, understanding the significance of the critical illness can prompt further discussions about the patient’s wishes for future medical treatment. For patients who deteriorate in the ICU, families are better prepared for bad news, rather than being ‘blindsided’ by an unexpected death in hospital.

Beyond these initial updates with a patient’s family, Australian intensivists typically hold formal family meetings if there is significant news to convey (for example, major deterioration or a worrying new test result), or an important decision to make (for example, whether to proceed to tracheostomy). Table 2 outlines the key principles guiding communication in family meetings [27, 28].

**Table 2:** Principles guiding communication in family meetings [27, 28]

Before the meeting • Ensure a comprehensive understanding of the patient’s clinical condition, including chart review and complete clinical examination • Speak with senior clinicals from other specialty teams caring for the patient to understand their clinical perspective and avoid presenting conflicting information to the family • ‘Pre-meet’ with the clinicians attending the meeting (at least one ICU doctor and one ICU nurse) and talk through planned discussion points
During the meeting • Focus on building trust and rapport • Talk less, listen more - allow families time to share their perspective • Seek to understand the patient’s values • Use simple phrases, avoid confusing terminology or jargon • Share relatable predictions of outcome rather than statistics or illness severity modelling
After the meeting • Check in with the clinical team • Document key discussion points to ensure continuity of communication for incoming clinicians

### What is good end of life care?

What is a ‘good death’? The answers to this question will vary between individuals, families, religions and cultures [35]. Nonetheless, common themes arise, and these can guide high-quality end of life care. While many people would not choose to die in an ICU (or even in hospital), clinicians can act to promote the patient’s comfort and dignity at the end of life, even within the highly technological clinical environment of the ICU [36].

Most importantly, to deliver good quality end of life care, it is essential to recognise that the patient is dying [37]. This should then be conveyed compassionately and clearly to the family, using simple language rather than euphemisms or medical jargon. Australian intensivists generally prefer to use the words ‘dying’ or ‘death’ rather than euphemisms, such as ‘passed on’. Recognising dying helps to ensure that clinicians do not inadvertently prolong a patient’s suffering by continuing burdensome treatment without hope of recovery.

Similarly, Australian Intensivists strive to recognise when treatment will not restore the patient to a life, he or she would find acceptable [38]. This requires an understanding of the patient’s own goals and values, rather than making assumptions about what constitutes an acceptable quality of life. If a patient had clearly shared with their family that they would not find living in a nursing home acceptable, and this is their most likely functional outcome following critical illness, the treating intensivist would be expected to communicate with the patient and/or family, recommending against the initiation or continuation of vital organ support.

Good-quality end of life care attends to the needs of the both the patient and their family. As much as possible, Australian ICUs allow families to spend time at the bedside with the patient. Intrusions are by minimised by avoiding unnecessary clinical interruptions. For patients dying in the ICU, nursing observations focus on the patient’s level of comfort, rather than standard vital sign monitoring. To this end, it is common to remove invasive monitoring and other devices. In many instances, nurses will de-activate the bedside monitoring screens and continue monitoring remotely at a central staff station. It is an important distinction that while *treatments* may be limited or withdrawn, *care* for the patient never is. Most deaths in Australian ICUs involve the withdrawal or limitation of artificial life supports. Families are provided with strong assurances that the cessation of life-prolonging measures is not a form of abandonment, but rather a change in treatment priorities to ensure comfort and dignity are paramount. “One-way” extubation, stopping non-beneficial medications, ceasing renal replacement therapy, avoidance of intravenous fluids, and removing artificial nutrition are standard practices during palliation in Australian ICUs. It is also usual to continue or initiate infusions of opiates and benzodiazepines where necessary to alleviate pain, dyspnoea, anxiety or terminal agitation, noting that the primary intention is to lessen suffering rather than to hasten the dying process. While palliative care clinicians do not generally become directly involve in the management of patients located in the ICU [39, 40], in circumstances where dying will take several days, it would be common to arrange transfer to a dedicated palliative care ward, where there is access to such services.

To preserve the possibility of organ donation for all patients who are identified as dying in the ICU, referrals are made routinely to the local organ donation authority to check donor registration status and to assess medical suitability [24]. Where the patient is likely medically suitable for organ donation, the option of organ donation is offered to the family in a collaborative conversation involving the ICU team and a clinician with additional specialist training in organ donation. These ‘family donation conversations’ aim to support the family to make a well-informed decision that consistent with the patient’s own wishes and values [41].

Conversely, it is instructive to consider the characteristics of poor end of life care in the ICU. These include a failure to recognise dying, leading to prolonged burdensome treatment for the patient and moral distress for the ICU clinicians providing the treatment. Poor communication between the ICU team and other treating teams can lead to confusion about the patient’s trajectory, treatment, and prognosis. Similarly, infrequent and/or inconsistent communication with the patient’s family can lead to confusion, shock or anger if the patient deteriorates.

### Epilogue–what happened to Mr A

*In a subsequent family meeting, the daughter shared the emotional difficulty of accepting her father’s condition. The team carefully explored her concerns, clarified the goals of care, and emphasised that treatment could continue in ways consistent with her father’s values, prioritising comfort, dignity, and avoidance of unnecessary suffering. Through this process, she gradually recognised that her father would not have wanted ongoing invasive support under these circumstances. The discussion was conducted with the involvement of senior clinicians and palliative care, allowing time for questions, sharing of perspectives, and reassurance that care would continue, albeit in a form aligned with her father’s preferences. With his family present, the team proceeded with a palliative extubation, continuing sedation and analgesia to ensure comfort. Vasopressors were ceased, and Mr A died peacefully soon after.*

## Future challenges

Despite the increasing age and illness severity for patients admitted to Australian ICUs, survival outcomes are very good and have improved over time [1, 42]. The expectations of patients, families and society have also evolved, such that fewer therapies are considered off limits on the basis of age, frailty or cost. Medical technologies now offer therapies that are increasingly personalised, safer and more effective, having a dramatic impact across a range of serious diseases. Examples such as less invasive procedures for structural heart disease and the rapid progress in highly specific treatments for malignancy are just two of many domains, where intensivists must adapt their approach to acknowledge expanded indications for critical care support. The impact of these changes is largely positive; however, intensivists are increasingly challenged by how to manage complications that will inevitably occur in patients who are at the margins of eligibility for complex interventions. Even in wealthy countries, resources are not unlimited, and medical practitioners have an important role in determining appropriate expenditure when it is not possible to fund every demand in healthcare. It may be that an approach emphasising a ‘time-limited’ trial of therapy with explicit ‘ceilings’ on treatment intensity is a pragmatic approach to these new paradigms [43]. Similarly, at the point of discharging a complex vulnerable survivor from ICU, it may be helpful to document that, in the event of serious deterioration, readmission to the ICU would be “context dependant”, based on the likelihood of the new problem to be effectively treatable and survival in state consistent with the patient’s values is possible. Intensivists will surely need to maintain an in-depth knowledge of relevant legal and ethical considerations, as well high-level communication skills for these approaches to be accepted by patients, families and clinical colleagues.

## Conclusion

ICU clinicians can make a considerable impact on the experience of dying for patients and families. Australian ICU practice is supported by legislation, ethical frameworks and guidelines developed by specialist societies. By prioritising a patient’s values and avoiding non-beneficial care that may prolong suffering, families are guided with respect, compassion and kindness. Australian intensivist care trainees are required to know relevant legal considerations, master key communication skills and understand important ethical principles to progress to specialist practice; these capabilities must be maintained and developed for the duration of their intensive care career.

## Data Availability

No datasets were generated or analysed during the current study.

## References

[1] Warrillow S, Raper R. The evolving role of intensive care in health care and society. Med J Aust. 2019;211(7):294-297.e1. 10.5694/mja2.50340. 31544249 10.5694/mja2.50340

[2] Wang S, Cai W, Sun Q, et al. Landscape of multiculturalism in Australia: tracking ethnic diversity and its relation with neighbourhood features in 2001–2021. Appl Geogr. 2023;160:103114. 10.1016/j.apgeog.2023.103114.

[3] The ethical advantage: the economic and social benefits of ethics to Australia. The Ethics Centre- Deloitte Access Economics; 2020.

[4] Lickiss JN. Approaching death in multicultural Australia. Med J Aust. 2003;179(S6):S14–6. 10.5694/j.1326-5377.2003.tb05569.x. 12964928 10.5694/j.1326-5377.2003.tb05569.x

[5] Allotey P, Reidpath D, Manderson L. Addressing cultural diversity in Australian health services. Health Promot J Austr. 2002;13(2):29–33.

[6] Jones C, Rotherham H, Udy A, et al. The relationship between administratively recorded ethnicity and outcomes for people admitted to Australian intensive care units with COVID-19. Aust Crit Care. 2025;38(4):101228. 10.1016/j.aucc.2025.101228. 40273709 10.1016/j.aucc.2025.101228

[7] Ogunyemi D, Thind BS, Teixeira A, et al. Integrating cultural humility into medical education using a structured and interactive workshop. Adv Med Educ Pract. 2024;15:575–83. 10.2147/amep.S460970. 38911069 10.2147/AMEP.S460970 PMC11193470

[8] Exploratory Analysis of Barriers to Palliative Care. Melbourne: Australian Government Department of Health. 2019. (https://www.health.gov.au/sites/default/files/documents/2020/01/exploratory-analysis-of-barriers-to-palliative-care-summary-policy-paper.pdf).

[9] Alsaeed T, Washington T, Xia B. Comprehensive analysis of Australia’s aged care system to inform policies for a sustainable future. Front Public Health. 2025;13:1525988. 10.3389/fpubh.2025.1525988. 40290496 10.3389/fpubh.2025.1525988 PMC12021627

[10] van Loon AM, Depla M, Hertogh C, Huisman M, Kok AAL. The disability paradox? Trajectories of well-being in older adults with functional decline. J Aging Health. 2023;35(1–2):125–37. 10.1177/08982643221108660. 35713401 10.1177/08982643221108660 PMC9755699

[11] Advance Care Planning Australia. Brisbane South Palliative Care Collaborative at Metro South Health. (www.advancecareplanning.org.au).

[12] Silvester W, Detering K. A programme for advance care planning for older people: Respecting Patient Choices. In: Van den Block L, Albers G, Martins Pereira S, Onwuteaka-Philipsen B, Pasman R, Deliens L, eds. Palliative care for older people: A public health perspective: Oxford University Press. 2015:0.

[13] Buck K, Nolte L, Sellars M, et al. Advance care directive prevalence among older Australians and associations with person-level predictors and quality indicators. Health Expect. 2021;24(4):1312–25. 10.1111/hex.13264. 33932311 10.1111/hex.13264 PMC8369087

[14] Jeong S, Barrett T, Ohr SO, Cleasby P, Davey R, David M. Prevalence of advance care planning practices among people with chronic diseases in hospital and community settings: a retrospective medical record audit. BMC Health Serv Res. 2021;21(1):303. 10.1186/s12913-021-06265-y. 33820535 10.1186/s12913-021-06265-y PMC8022421

[15] Ducharlet K, Degen D, McMahon LP. Advance care planning: we need to change our approach to improve patient and clinician engagement. Kidney Int Rep. 2026;11(2):103702. 10.1016/j.ekir.2025.11.027. 41537101 10.1016/j.ekir.2025.11.027 PMC12796573

[16] Advance Care Planning Prevalence in Australia 2025. Brisbane: Advance Care Planning Australia. 2025.

[17] Carter RZ, Detering KM, Silvester W, Sutton E. Advance care planning in Australia: what does the law say? Aust Health Rev. 2016;40(4):405–14. 10.1071/ah15120. 26567895 10.1071/AH15120

[18] Detering KM, Hancock AD, Reade MC, Silvester W. The impact of advance care planning on end of life care in elderly patients: randomised controlled trial. BMJ. 2010;340:c1345. 10.1136/bmj.c1345. 20332506 10.1136/bmj.c1345 PMC2844949

[19] The Australian and New Zealand Intensive Care Society Statement on Death and Organ Donation. 4.1 ed. Melbourne: ANZICS. 2021.

[20] Informed consent to medical treatment. Melbourne: Australian Law Reform Commission. 2014.

[21] Del Villar K, Willmott L, White BP. Voluntary assisted dying-Australia in an international context. Med Law Rev. 2025. 10.1093/medlaw/fwaf025. 40708257 10.1093/medlaw/fwaf025 PMC12289548

[22] A guide to the Medical Treatment Planning and Decisions Act 2016. 2nd ed. Melbourne: Victorian Government. 2019.

[23] Statement on Withholding and Withdrawing Treatment. Melbourne: College of Intensive Care Medicine and Australian and New Zealand Intensive Care Society. 2023.

[24] Australia and New Zealand Intensive Care Society. Care and Decision-Making at the End of Life for the Critically Ill. Melbourne. 2014. (https://www.anzics.org/wp-content/uploads/2018/08/ANZICS-Statement-on-Care-and-Decision-Making-at-the-End-of-Life-for-the-Critically-Ill.pdf).

[25] Krnjacki L, Priest N, Aitken Z, et al. Disability‐based discrimination and health: findings from an Australian‐based population study. Aust N Z J Public Health. 2018;42(2):172–4. 10.1111/1753-6405.12735. 29168323 10.1111/1753-6405.12735

[26] College of Intensive Care of Australia and New Zealand. What is an Intensive Care Specialist?. Melbourne, Australia. 2025. (December 2025) (https://cicm.org.au/Web/Web/AboutUs/What-is-an-Intensive-Care-Specialist.aspx?hkey=fcfa6d83-9eef-491b-ac90-ec98c827f84a).

[27] Warrillow S, Farley KJ, Jones D. Ten practical strategies for effective communication with relatives of ICU patients. Intensive Care Med. 2015;41(12):2173–6. 10.1007/s00134-015-3815-0. 25904186 10.1007/s00134-015-3815-0

[28] Warrillow S, Farley KJ, Jones D. How to improve communication quality with patients and relatives in the ICU. Minerva Anestesiol. 2016;82(7):797–803. 26883747

[29] CPD Home- Overview and Requirements. College of Intensive Care Medicine of Australia and New Zealand (https://cicm.org.au/Web/Web/CPD/Overview-and-Requirements.aspx).

[30] Bloomer MJ, Ranse K, Butler A, Brooks L. A national position statement on adult end-of-life care in critical care. Aust Crit Care. 2022;35(4):480–7. 10.1016/j.aucc.2021.06.006. 34384650 10.1016/j.aucc.2021.06.006

[31] Brooks LA, Bloomer MJ, Manias E. Culturally sensitive communication at the end-of-life in the intensive care unit: a systematic review. Aust Crit Care. 2019;32(6):516–23. 10.1016/j.aucc.2018.07.003. 30237059 10.1016/j.aucc.2018.07.003

[32] Bellomo R, Stow PJ, Hart GK. Why is there such a difference in outcome between Australian intensive care units and others? Curr Opin Anaesthesiol. 2007;20(2):100–5. 10.1097/ACO.0b013e32802c7cd5. 17413391 10.1097/ACO.0b013e32802c7cd5

[33] Cleeve B, Fielding R, Salonga S, Guille C, Barrett J. ICU multidisciplinary team meetings improve communication, discipline profile and patient care. Aust Crit Care. 2020;33:S38. 10.1016/j.aucc.2020.04.120.

[34] Hampton JM, Ward EC, Morrison L, et al. The development and feasibility of a psychologist-led screening and modular-based psychological intervention in an Australian intensive care unit: a pilot study. Aust Crit Care. 2025. 10.1016/j.aucc.2025.101301. 40779818 10.1016/j.aucc.2025.101301

[35] Krikorian A, Maldonado C, Pastrana T. Patient’s perspectives on the notion of a good death: a systematic review of the literature. J Pain Symptom Manage. 2020;59(1):152–64. 10.1016/j.jpainsymman.2019.07.033. 31404643 10.1016/j.jpainsymman.2019.07.033

[36] Lovell T, Mitchell M, Powell M, et al. An interprofessional multicomponent intervention to improve end-of-life care in intensive care: a before-and-after study. Aust Crit Care. 2025;38(3):101147. 10.1016/j.aucc.2024.101147. 39689996 10.1016/j.aucc.2024.101147

[37] Care of Dying Adults in the Last Days of Life. National Institute for Health and Care Excellence (NICE). (https://www.ncbi.nlm.nih.gov/books/NBK356012/).

[38] ANZICS Statement on Care and Decision-Making at the End of Life for the Critically Ill. 1.0 ed. Melbourne: Australian and New Zealand Intensive Care Society. 2014.

[39] Bloomer MJ, Tiruvoipati R, Tsiripillis M, Botha JA. End of life management of adult patients in an Australian metropolitan intensive care unit: a retrospective observational study. Aust Crit Care. 2010;23(1):13–9. 10.1016/j.aucc.2009.10.002. 19914844 10.1016/j.aucc.2009.10.002

[40] Bone A, Elderkin T. Palliative care practice in the intensive care unit: perceptions of registered nurses. Aust Crit Care. 2010;23(1):39. 10.1016/j.aucc.2009.12.022.

[41] O’Leary M, Mark M, Elena C. 323.7: Participation of a donation nurse in family donation conversations is associated with higher rates of family consent to donation irrespective of who leads the conversation. Transplantation. 2025;109(12S):S95. 10.1097/01.tp.0001175300.23757.1b.

[42] Pilowsky JK, Ueno R, McLarty J, Pilcher D, Bailey M, Brown A. Mortality trends across key diagnostic groups in Australian and New Zealand ICUs over the past 30 years. Crit Care Med. 2025;53(11):e2124–33. 10.1097/ccm.0000000000006817. 40810586 10.1097/CCM.0000000000006817 PMC12577652

[43] Kruser JM, Ashana DC, Courtright KR, et al. Defining the time-limited trial for patients with critical illness: an official American Thoracic Society workshop report. Ann Am Thorac Soc. 2024;21(2):187–99. 10.1513/AnnalsATS.202310-925ST. 38063572 10.1513/AnnalsATS.202310-925ST PMC10848901

